# Effect of Incorporating Hydroxyapatite and Zinc Oxide Nanoparticles on the Compressive Strength of White Mineral Trioxide Aggregate

**DOI:** 10.30476/DENTJODS.2020.82963.1034

**Published:** 2020-12

**Authors:** Mahsa Eskandarinezhad, Mostafa Ghodrati, Fateme Pournaghi Azar, Farnaz Jafari, Parvin Samadi Pakchin, Amir Ardalan Abdollahi, Amir Houman Sadrhaghighi, Forouzan Rezvan

**Affiliations:** 1 Dept. of Endodontics, Dental and Periodontal Research Center, Dental School, Tabriz University of Medical Sciences, Tabriz, Iran; 2 Dental and Periodontal Research Center, Dept. of Operative Dentistry, Faculty of Dentistry, Tabriz University of Medical Sciences, Tabriz, Iran; 3 Research Center for Pharmaceutical Nanotechnology, Tabriz University of Medical Sciences, Tabriz, Iran; 4 Dept. of Endodontics, Dental School, Urmia University of Medical Sciences, Urmia, Iran; 5 Dept. of Orthodontics, Faculty of Dentistry, Tabriz University of Medical Science, Tabriz, Iran; 6 Private Practice, Tabriz, Iran

**Keywords:** Compressive strength, Hydroxyapatite, Mineral trioxide aggregate, Nanoparticles

## Abstract

**Statement of the Problem:**

Many efforts have been made to improve the properties of mineral trioxide aggregate (MTA), including the incorporation of nanoparticles.

**Purpose:**

The aim of this study was to investigate the incorporation of zinc oxide and hydroxyapatite nanoparticles on the compressive strength of white MTA (WMTA).

**Materials and Method:**

In this in vitro study, the following materials were evaluated: MTA, MTA+5% zinc oxide (ZnO) nanoparticles, MTA+10% zinc oxide nanoparticles, MTA+5% hydroxyapatite (HA) nanoparticles, MTA+10% zinc oxide nanoparticles. The compressive strength of the groups under investigation was measured on days 4 and 21 after mixing the MTA using a universal testing machine. Two-way ANOVA test was used to compare the groups and determine the significance of the effect of time and material on the compressive strength (*p*<0.05).

**Results:**

The highest and lowest compressive strength values were respectively measured for the second group, MTA/21 days, and the fourth group, MTA+Nano ZnO/4 days. Two-way ANOVA indicated that incorporation of zinc oxide and hydroxyapatite nanoparticles into MTA did not have a significant effect on compressive strength (*p*= 0.05). Compressive strength in all the groups increased over time from day 4 to day 21. However, this increase was not statistically significant (*p*= 0.06) except for the MTA group, which exhibited significant increase in compressive strength over time from day 4 to day 21 (*p*=0.007).

**Conclusion:**

Incorporation of HA and ZnO nanoparticles into MTA had no detrimental effects on its strength and these nanoparticles can be used to improve the other properties of MTA.

## Introduction

Mineral trioxide aggregate (MTA) is hydrophilic cement with calcium silicate base, which is widely used in endodontics to repair perforations, in vital pulp therapy and as a surgical retrograde material due to several favorable properties, including biocompatibility and the ability to induce osteogenesis and cementogenesis [ 1 - 3 ]. One of the disadvantages of MTA is its long setting time and difficult handling [ 4 - 5 ]. Various methods have been proposed to improve the MTA properties, including adding materials such as sodium hydrogen phospha-te (Na2HPO4), calcium chloride, and nanoparticles [ 6 - 9 ].

The physical properties of an endodontic biomaterial, such as compressive strength, are important in cases where this material is subjected to occlusal forces [ 10 ]. The similar clinical conditions of this case are the application of MTA as a pulp capping material, in apexogenesis and in perforation repair [ 11 ], where the material is subjected to the force of restorative materials as well as to occlusal forces [ 12 ]. According to previous studies, the compressive strength for MTA immediately after setting is 40 MPa (Mega Pascal), which increases to 67 MPa within 21 days [ 13 - 14 ]. Several factors affect the compressive strength, including the type of MTA, condensation pressure, the acid etching process, the mixing technique, and the liquid mixed with MTA [ 12 - 13 ]. Over time, various materials have been added to improve the properties of MTA. One of the materials that have been considered in this regard in various studies is nanoparticles, including silica, silver, and silver zeolite [ 9 , 15 - 17 ]. In one study, the incorporation of silica nanoparticles reduced the setting time and increased the compressive and flexural strengths of MTA [ 16 ].

Hydroxyapatite (HA) is an important biological material and is the main component of the mineral bone and teeth [ 18 ]. It is widely used in medicine and dentistry [ 18 - 19 ]. HA is used to improve the setting time of MTA [ 20 ] and to improve the osteogenic properties because of its biocompatibility [ 21 ].

Zinc oxide (ZnO) particles are antimicrobial substances, which have been used for many years in various dental compositions due to biological adaptation [ 17 ]. Zinc activates enzymes, which are toxic to bacteria at low concentrations, and inhibits plaque growth at higher concentrations [ 22 ].

The effect of adding new agents to MTA mixture on its properties should be assessed and so far there is no published study on the compressive strength of MTA mixed with ZnO and HA nanoparticles. This study was designed to evaluate the properties of this substance after 4 and 21 days.

## Materials and Method

### Determination of the sample size

The study was approved by the Research and Ethics Committee of Tabriz University of Medical Sciences. A sample size of 64 was considered based on the results of a pilot study, the materials used and the evaluation time of the compressive strength (8 groups, n=8). The study groups mentioned in Table 1 was based on the type of the material in the cylinder and the evaluation time of the compressive strength test.

**Table 1:** Study groups

Study groups	Type of material inside cylinder	Evaluation time
1	MTA	4
2	MTA	21
3	10% Nano ZnO+MTA	4
4	10% Nano ZnO+MTA	21
5	10% Nano HA+MTA	4
6	10% Nano HA+MTA	21
7	5% Nano HA+5% Nano ZnO+MTA	4
8	5% Nano HA+5% Nano ZnO+MTA	21

In this study, we considered that all the cylinders were filled with the material and that the upper surface of the material was leveled with the edges of the cylinder. The cylinders that did not have this feature were excluded from the study.

### Confounding variables

Since the setting condition of the material affects the compressive strength, the conditions for mixing and placing the materials and the test time were considered the same for all the specimens. In addition, the materials were placed inside the cylinders and mixed by one operator. The condenser size used to pack the material inside the cylinder was also the same for all the specimens. The powder-to-liquid ratio of 3:1 was the same in all the samples. The whole stages of the work were based on the ISO standard, which minimizes the probability of error.

### Procedural steps

The HA powder was purchased from SIGMA-ALDRICH Co. (USA). ZnO nanoparticles were synthesized by a nano-technologist colleague in Tehran University of Medical Sciences using Zn(CH3COO)2.2H2O and NaOH with methanol solvent. The resultant mixture was transferred to an autoclave and kept at 120°C for 6 hours. The solid and white product was then isolated by filtering. The crystal structure of nanoparticles was examined by x-ray diffraction (XRD) and the morphology of nanoparticle was examined under a scanning electron microscope (SEM). The amount of surface hydrophilic rate was examined by contact angle test and particle size was examined by dynamic light scattering (DLS). In groups 1 and 2, white ProRoot MTA powder (Dentsply, Tulsa, USA) was mixed with physiologic serum at a powder-to-liquid ratio of 3:1 according to the manufacturer's instructions and was placed within the molds within thirty seconds after mixing. According to the standard, the selected stainless steel molds had a height of 6 mm and a diameter of 3 mm, the internal surface of which was lubricated with paraffin. In groups 3 and 4, 10% Nano ZnO+MTA powder was used. First, MTA powder weight (wt) was measured using a digital weighing machine and ZnO was added at 10 wt% of it. In the remaining groups, the weight of the nanoparticles was calculated with this method and added to MTA; groups 5 and 6, 10% Nano HA+MTA and in groups 7 and 8, 5% Nano HA+5% Nano ZnO+MTA were mixed. The powders of the above groups were mixed with a ratio of 3:1 with the serum, transferred to the molds using a MTA carrier and packed into the selected molds with a dental plugger. The samples were then welded in distilled water gas and stored in a container at 37°C until the compressive strength measurement.

The compressive strength was measured accordance to ISO 6876. The device used for this purpose was a universal testing machine (Hounsfield Test Equipment, model: H5K-S, Perrywood Business Park, Honey Corckland, Salfords, Redhill, Surrey, UK).

The samples in groups 1, 3, 5 and 7 were evaluated on the 4th day, and the samples in groups 2, 4, 6 and 8 were evaluated on the 21st day. The samples were retrieved from the molds and the machine head applied a force at a speed of 1 mm/min on the longitudinal axis until the material was crushed or broken. This force was registered in Newton and converted to MPa with the formula of CS = 4p/μd, where p is the maximum force applied in Newton, and d is the actual diameter of the samples in mm.

### Statistical analysis

Statistical analysis was performed using SPSS (Statistical Package for Social Science, SPSS, version 20.0, SPSS, Chicago, IL, USA).The Kolmogorov-Smirnov test was used to check the normal distribution of the data. After calculating the mean ± standard deviation of compressive strength, two-way ANOVA test was used to compare the groups and determine the significance of the effect of time and material on the compressive strength. In the current study, *p*< 0.05 was considered statistically significant.

## Results

The results of the descriptive statistics, i.e. means and standard deviations of the data (Table 2) showed the highest compressive strength at 4-day interval after mixing for the MTA+Nano HA group (38.5±9.24) and the lowest compressive strength at this time interval for MTA+Nano ZnO group (26.5±9.42).

**Table 2:** Means and standard deviations of the compressive strength of the study groups

Groups	Compressive strength 4 days after mixing (MPa)	Compressive strength 21 days after mixing (MPa)	*p* Value
MTA	31.12±6.19	41.12±6.53	0.007*
MTA+Nano ZnO	26.5±9.42	33.5±10.4	0.180
MTA+Nano HA	38.5±9.24	40±12.66	0.882
MTA+Nano ZnO+ Nano HA	26.87±6.49	30.75±6.54	0.254

* p< 0.05 and the difference between the two groups is significant.

At 21-day interval after mixing, the highest compressive strength was recorded in the MTA group (41.12±6.53), with the lowest value in the MTA+Nano ZnO + Nano HA group (30.75±6.54).

In total, the highest compressive strength (41.12± 6.53 MPa) and the lowest compressive strength (26.5± 9.42 MPa) were measured in the second group (MTA/ 21 days) and the fourth group (MTA+Nano ZnO/4 days), respectively.

Two-way ANOVA showed that (Table 3) incorporation of zinc oxide and HA nanoparticles into MTA did not have a significant effect on its compressive strength (*p*= 0.05).

**Table 3:** The results of two-way ANOVA

	*p* Value
Group	0.50
Time	0.067
Group–Time	0.766

**Figure 1:**
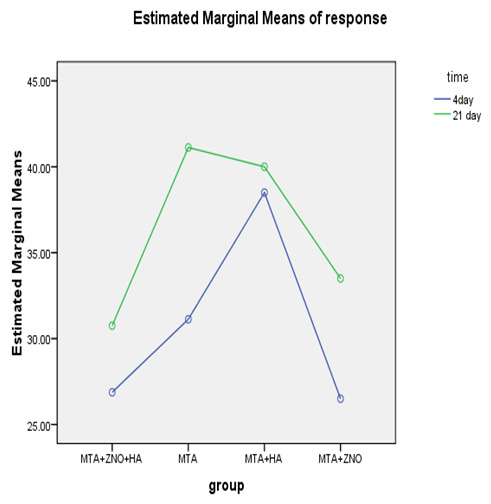
Compressive strength of different groups at different time intervals.

It should be noted that incorporation of Nano HA into MTA 4 days after mixing increased compressive strength compared to pure MTA, which was not statistically significant (*p*> 0.05). Moreover, incorporation of Nano ZnO also reduced the compressive strength at both time intervals, but the difference was not statistically significant (*p*> 0.05). It was also found that in all the groups, the compressive strength increased from day 4 to day 21 but this increase was not statistically significant (*p*= 0.06), except for the MTA group where the compressive strength increased significantly from day 4 to day 21(*p*= 0.007) (Figure 1).

## Discussion

MTA is a calcium silicate-based material. It is highly biocompatible and can induce hard tissue formation due to its favorable characteristics. It has been accepted as the best standard for endodontic treatments for many years [ 23 - 24 ]. In spite of these desirable characteristics, long setting time and technical sensitivity of this material have prompted the researchers to add different materials, including nanoparticles, to improve some of its properties. Considering its application in the field of root canal treatment, its physical properties, including compressive strength, have been of great importance in various studies [ 13 , 25 - 26 ], which should be re-examined after adding any material to its structure. The initial strength of MTA is 40 MPa and according to previous studies, it reaches 67 MPa in 21 days [ 14 , 27 ]. Compressive strength reflects the quality of the hydration process and hydration is a factor directly affecting the MTA setting; therefore, any factor affecting the hydration process also affects its physical properties [ 2 , 10 , 25 , 28 - 29 ]. 

One of the materials evaluated in this study was HA, which is considered as an important biological material and the main component of the bone and teeth. The HA nanoparticles are more efficient because of fine particles, having more contact surfaces and higher solubility [ 30 ]. The HA has bone formation potential and can directly bind to the bone [ 31 ]. It is easily tolerated and integrated into the host tissue [ 32 ] and because of the lack of protein, it does not cause allergic and immune reactions [ 33 ].

In the current study, incorporation of HA nanoparticles had no significant effect on MTA compressive strength, and considering the favorable properties of this nanoparticle, this material can be used to improve other properties of MTA without reducing its compressive strength. On the fourth day after mixing, the compressive strength of the group, where HA was added to MTA, was higher than the MTA group alone, which not statistically significant.

HA is a highly active substance that results in rapid bonding due to its nano-structure and reduces the setting time [ 34 ], which can increase compressive strength on the early days (4 days after mixing).

In other studies, various nanoparticles have been added to improve the properties of MTA [ 6 , 15 - 16 ]. In a study by Akbari *et al*. [ 16 ], it was found that incorporation of nano silicate particles such as the particles used in our study had a positive effect on compressive strength of MTA, but this effect was not significant.

A study by Prasad *et al*. [ 6 ] showed that incorporation of calcium formate, calcium chloride, and di-sodium hydrogen orthophosphate nanoparticles, in contrast to the nanoparticles used in our study, reduced the compressive strength of MTA. In a study by Bernardi *et al*. [ 26 ], calcium carbonate nanoparticles reduced the compressive strength of MTA. Another nanoparticle considered in our study was ZnO, which has been shown in many studies to have antibacterial properties [ 22 , 35 ]. 

In the group in which ZnO alone was added to MTA, the compressive strength slightly decreased although this decrease was not statistically significant. However, in a study by Guerreiro-Tanomaru *et al*. [ 36 ], incorporation of ZnO into calcium silicate-based materials (such as MTA) resulted in a significant reduction in compressive strength. This might be due to the reaction effect of these nanoparticles with MTA due to cracks in the MTA structure, explaining the slight decrease in the compressive strength of the MTA and ZnO groups. In a study by Samiei *et al*. [ 15 ], incorporation of silver-zinc-zeolite nanoparticles to MTA caused a significant reduction in compressive strength. The reason for this decrease in compressive strength of MTA after adding some materials is the impact on the MTA hydration and setting process.

We selected two time intervals for measuring the compressive strength in this study. The shorter time of 4 days was selected because the initial strength is important in clinical applications and the material is initially exposed in the patient's mouth to occlusal forces. Therefore, the material in this period undergoes favorable setting; moreover, the same period has been selected for this purpose in previous studies [ 15 , 28 ].

The 21-day period was also selected to study the effects of HA and ZnO in a longer period and along with a shorter period, the results were compared with the control group (without nanoparticles). Long-term strength is important for materials’ resistance to occlusal force and the force created by the placement of the restorative materials [ 28 ].

A significant increase in compressive strength was observed over time from day 4 to day 21 only in the MTA group alone, which is consistent with previous studies [ 15 , 26 ]. In other groups, there was a slight increase in the compressive strength, which was not statistically significant.

## Conclusion

According to the results of the present study, HA and ZnO nanoparticles had no significant effects on the compressive strength of MTA. Therefore, regarding the advantages of these nanoparticles, they can be employed either in cases where compressive strength is important such as the repair of furcal perforations, pulp capping and apexogenesis, or in the case of apical plug and as a retrograde material in surgery where the compressive strength is not important.

## References

[1] Lotfi M, Ghasemi N, Rahimi S, Bahari M, Vosoughhosseini S, Saghiri MA, et al ( 2014). Effect of smear layer on the push-out bond strength of two endodontic biomaterials to radicular dentin. Iran Endod J.

[2] Aminabadi NA, Huang B, Samiei M, Agheli S, Jamali Z, Shirazi S ( 2016). A Randomized Trial Using 3Mixtatin Compared to MTA in Primary Molars with Inflammatory Root Resorption: A Novel Endodontic Biomaterial. J Clin Pediatr Dent.

[3] Asl Aminabadi N, Satrab S, Najafpour E, Samiei M, Jamali Z, Shirazi S ( 2016). A randomized trial of direct pulp capping in primary molars using MTA compared to 3Mixtatin: a novel pulp capping biomaterial. Int J Paediatr Dent.

[4] Ghasemi N, Rahimi S, Lotfi M, Solaimanirad J, Shahi S, Shafaie H, et al ( 2014). Effect of Mineral Trioxide Aggregate, Calcium-Enriched Mixture Cement and Mineral Trioxide Aggregate with Disodium Hydrogen Phosphate on BMP-2 Production. Iran Endod J.

[5] Zapf AM, Chedella SC, Berzins DW ( 2015). Effect of additives on mineral trioxide aggregate setting reaction product formation. J Endod.

[6] Prasad A, Pushpa S, Arunagiri D, Sawhny A, Misra A, Sujatha R ( 2015). A comparative evaluation of the effect of various additives on selected physical properties of white mineral trioxide aggregate. J Conserv Dent.

[7] Samiei M, Aghazadeh M, Lotfi M, Shakoei S, Aghazadeh Z, Vahid Pakdel SM ( 2013). Antimicrobial Efficacy of Mineral Trioxide Aggregate with and without Silver Nanoparticles. Iran Endod J.

[8] Eskandarinezhad M, Shahveghar-Asl N, Sharghi R, Shirazi S, Shakouie S, Milani AS, et al ( 2017). Sealing efficacy of mineral trioxide aggregate with and without nanosilver for root end filling: An in vitro bacterial leakage study. J Clin Exp Dent.

[9] Samiei M, Janani M, Asl-Aminabadi N, Ghasemi N, Divband B, Shirazi S, et al ( 2017). Effect of the TiO2 nanoparticles on the selected physical properties of mineral trioxide aggregate. J Clin Exp Dent.

[10] Parirokh M, Torabinejad M ( 2010). Mineral trioxide aggregate: a comprehensive literature review--Part III: Clinical applications, drawbacks, and mechanism of action. J Endod.

[11] Baroudi K, Samir S ( 2016). Sealing Ability of MTA Used in Perforation Repair of Permanent Teeth; Literature Review. Open Dent J.

[12] Shahi S, Ghasemi N, Rahimi S, Yavari HR, Samiei M, Janani M, et al ( 2015). The Effect of Different Mixing Methods on the pH and Solubility of Mineral Trioxide Aggregate and Calcium-Enriched Mixture. Iran Endod J.

[13] Sobhnamayan F, Adl A, Shojaee NS, Sedigh-Shams M, Zarghami E ( 2017). Compressive Strength of Mineral Trioxide Aggregate and Calcium-enriched Mixture Cement Mixed with Propylene Glycol. Iran Endod J.

[14] Parirokh M, Torabinejad M ( 2010). Mineral trioxide aggregate: a comprehensive literature review--Part I: chemical, physical, and antibacterial properties. J Endod.

[15] Samiei M, Ghasemi N, Asl-Aminabadi N, Divband B, Golparvar-Dashti Y, Shirazi S ( 2017). Zeolite-silver-zinc nanoparticles: Biocompatibility and their effect on the compressive strength of mineral trioxide aggregate. J Clin Exp Dent.

[16] Akbari M, Zebarjad SM, Nategh B, Rouhani A ( 2013). Effect of nano silica on setting time and physical properties of mineral trioxide aggregate. J Endod.

[17] Samiei M, Torab A, Hosseini O, Abbasi T, Abdollahi AA, Divband B ( 2018). Antibacterial Effect of Two Nano Zinc Oxide Gel Preparations Compared to Calcium Hydroxide and Chlorhexidine Mixture. Iran Endod J.

[18] Kattimani VS, Prathigudupu RS, Jairaj A, Khader MA, Rajeev K, Khader AA ( 2019). Role of Synthetic Hydroxyapatite-In Socket Preservation: A Systematic Review and Meta-analysis. J Contemp Dent Pract.

[19] Mohammadi Z, Shalavi S ( 2011). Effect of Hydroxyapatite and Bovine Serum Albumin on the Antibacterial Activity of MTA. Iran Endod J.

[20] Opacic-Galic V, Petrovic V, Zivkovic S, Jokanovic V, Nikolic B, Knezevic-Vukcevic J, et al ( 2013). New nanostructural biomaterials based on active silicate systems and hydroxyapatite: characterization and genotoxicity in human peripheral blood lymphocytes. Int Endod J.

[21] Van Esterik FA, Zandieh-Doulabi B, Kleverlaan CJ, Klein-Nulend J ( 2016). Enhanced Osteogenic and Vasculogenic Differentiation Potential of Human Adipose Stem Cells on Biphasic Calcium Phosphate Scaffolds in Fibrin Gels. Stem Cells Int.

[22] Spencer CG, Campbell PM, Buschang PH, Cai J, Honeyman AL ( 2009). Antimicrobial effects of zinc oxide in an orthodontic bonding agent. Angle Orthod.

[23] Moretton TR, Brown CE, Legan JJ, Kafrawy AH ( 2000). Tissue reactions after subcutaneous and intraosseous implantation of mineral trioxide aggregate and ethoxybenzoic acid cement. J Biomed Mater Res.

[24] Parirokh M, Mirsoltani B, Raoof M, Tabrizchi H, Haghdoost AA ( 2011). Comparative study of subcutaneous tissue responses to a novel root-end filling material and white and grey mineral trioxide aggregate. Int Endod J.

[25] Bidar M, Eslami N, Naghavi N, Fasihi Z, Attaran Mashhadi N ( 2015). The effect of different concentrations of chlorhexidine gluconate on the compressive strength of mineral trioxide aggregate. J Dent Res Dent Clin Dent Prospects.

[26] Bernardi A, Bortoluzzi EA, Felippe WT, Felippe MC, Wan WS, Teixeira CS ( 2017). Effects of the addition of nanoparticulate calcium carbonate on setting time, dimensional change, compressive strength, solubility and pH of MTA. Int Endod J.

[27] Nielsen MJ, Casey JA, VanderWeele RA, Vandewalle KS ( 2016). Mechanical properties of new dental pulp-capping materials. Gen Dent.

[28] Shahi S, Ghasemi N, Rahimi S, Yavari H, Janani M, Mokhtari H, et al ( 2015). The Effect of Different Mixing Methods on Working Time, Setting Time, Dimensional Changes and Film Thickness of Mineral Trioxide Aggregate and Calcium-Enriched Mixture. Iran Endod J.

[29] Salem-Milani A, Ghasemi S, Rahimi S, Ardalan-Abdollahi A, Asghari-Jafarabadi M ( 2017). The Discoloration effect of White Mineral Trioxide Aggregate (WMTA), Calcium Enriched Mixture (CEM), and Portland Cement (PC) on Human Teeth. J Clin Exp Dent.

[30] Thein-Han WW, Shah J, Misra RD ( 2009). Superior in vitro biological response and mechanical properties of an implantable nanostructured biomaterial: Nanohydroxyapatite-silicone rubber composite. Acta Biomater.

[31] Pradeep K, Kudva A, Narayanamoorthy V, Cariappa KM, Saraswathi MV ( 2016). Platelet-rich fibrin combined with synthetic nanocrystalline hydroxy apatite granules in the management of radicular cyst. Niger J Clin Pract.

[32] Suwanprateeb J, Thammarakcharoen F, Wasoontararat K, Chokevivat W, Phanphiriya P ( 2012). Single step preparation of nanosilver loaded calcium phosphate by low temperature co-conversion process. J Mater Sci Mater Med.

[33] Ogose A, Hotta T, Kawashima H, Kondo N, Gu W, Kamura T, et al ( 2005). Comparison of hydroxyapatite and beta tricalcium phosphate as bone substitutes after excision of bone tumors. J Biomed Mater Res B Appl Biomater.

[34] Jokanovic V, Jokanovic B, Izvonar D, Dacic B ( 2008). Thin films of SiO2 and hydroxyapatite on titanium deposited by spray pyrolysis. J Mater Sci Mater Med.

[35] Aydin Sevinc B, Hanley L ( 2010). Antibacterial activity of dental composites containing zinc oxide nanoparticles. J Biomed Mater Res B Appl Biomater.

[36] Guerreiro-Tanomaru JM, Trindade-Junior A, Costa BC, da Silva GF, Drullis Cifali L, Basso Bernardi MI, et al ( 2014). Effect of zirconium oxide and zinc oxide nanoparticles on physicochemical properties and antibiofilm activity of a calcium silicate-based material. Scientific World Journal.

